# Normal Labor

**Published:** 1897-02

**Authors:** J. W. Cole

**Affiliations:** Kennedale, Texas


For the Texas Medical Journal.

### NORMAL LABOR.

BY J. W. COLE, M. D., KENNEDALE, TEXAS.

I HAVE seen a great deal written lately about the best mode of conducting a normal childbirth. I notice some physicians handle these cases as though it was a diseased condition, giving this and that, and injecting this and that solution. Now, in my humble judgment, this is all unnecessary. When a patron speaks to me to wait on his wife in confinement, I inquire into the condition of her bowels, and if I find her constipated, I order some mild purgative, such as syrup of figs, aro. syrup rhei or fluid ext. cas. sag., or something on this line. I also instruct them to use an enema as soon as they find labor is coming on. I do this to work out the lower bowels, and with these simple instructions I leave the case to nature until I am called to wait on the woman in labor. On my arrival I inquire whether or not my instructions have been carried out, and if I find her bowels have not acted, I at once have the bowels washed out. I have often seen my hands full of hard fecal lumps passed off with the water. If she is having a pain now and then, I call for warm water and soap, and wash my hands. I never use a disinfectant, but I do not object to them. After washing my hands, I oil them well with oil or lard, and make an examination. I try to fully satisfy myself as to the position of the child, and also the condition of the soft parts, and when I find everything all right—position natural, soft parts dilating—I usually rupture the membranes. I then encourage her all I can, telling her everything is as it should be, and for her to be patient, and everything will come off in a few hours to our satisfaction.

I rarely use chloroform; never use ergot until the womb is empty. I sometimes, in very long, tedious cases, use a little chloroform. At the conclusion of the second stage of labor, I give my patient ℨi. of fluid ext., ergot and free dose of whisky or brandy. While one of my assistants is doing this, I see that the little fellow is all right, inflating his lungs if necessary. I then tie the cord with a nice clean string which I make from a ball of cotton thread. I usually tie once, but sometimes tie twice. After I cut the cord, I dress it in absorbent cotton, drawing it moderately tight with the band.

After ten or fifteen minutes rest, if the placenta has not been expelled, I can generally cause its expulsion by gently drawing the cord with my right hand, and with the left hand I firmly grasp the womb from above, and use some little force pressing it downward and backward. In this way I usually cause its expulsion within five or ten minutes. Now, then, at the close of the third stage of labor, I remove, as near as is possible, every damp garment, wrap the patient in dry, warm clothes and blankets. I do not use a bandage at all, but I insist on the woman's remaining in bed for two weeks. I always give a prescription for a bottle of carbolized olive oil, and have the soft parts well oiled with this twice daily. I usually leave a few doses of antikamnia to relieve after-pains, should they become troublesome; with this exception, I leave no medicine. I have been practicing medicine sixteen years in this, and an adjoining county, and I have waited on nearly 600 women, and I have never had a single woman to die from blood poison. I have had only seven still-born children, and five of these I was able to resuscitate. I delivered four children that had been dead some time. I have never lost a single woman directly from the effects of confinement. I consider the using of a syringe by unskilled hands, after delivery, very dangerous. There are so many ways by which harm may come, that I never allow a syringe used. Should it be necessary, I will use it myself. Keep my patient clean, and nature does the balance.

